# Supplementary material for: Super‐beacons: Open‐source probes with spontaneous tuneable blinking compatible with live‐cell super‐resolution microscopy
Source: Traffic. 2020 Mar 30;21(5):375–85. doi: 10.1111/tra.12728 (PMC7643006; doi:10.1111/tra.12728)
Supplement: Supplementary file 1 — FIGURE S1 Super‐beacons structural, thermal and chemical tunability. A, On time ratio r = τON/τOFF, against illumination intensity, where τON and τOFF are the weighted means of the on‐ and dark‐state distributions, respectively. B3S9L‐ATTO647N‐BHQ2 (red line). B3S9L‐ATTO647N (black line). Solid lines correspond to imaging performed in PBS; dashed lines correspond to imaging performed in switching buffer OxEA. B, On time ratio r = τON/τOFF, against illumination intensity, where τON and τOFF are the weighted means of the on‐ and dark‐state distributions, respectively. B3S9L‐ATTO647N‐BHQ2 (red line). B3S9L‐ATTO647N (black line). B3S9L‐ATTO647N‐BHQ2 (green line). Imaging performed in MiliQ water at 23°C. C, Same as in (B) but imaging performed in PBS at 37°C. Figure S2. Fourier Ring Correlation (FRC) resolution mapping and NanoJ‐SQUIRREL error maps of images acquired using SB or control probe. (Left) β‐tubulin immunolabelling with control probe (B3S9L‐ATTO555): WF snapshots (WF), super‐resolution renderings (STORM), NanoJ‐SQUIRREL error map of intensity normalised WF and STORM images (Error map), equivalent FRC map (FRC map), overlay between STORM and FRC map (Overlay). (Right) β‐tubulin immunolabelling with B3S9L SB probe (B3S9L‐ATTO555‐BHQ2): WF snapshots (WF), super‐resolution renderings (STORM), NanoJ‐SQUIRREL error map of intensity normalised WF and STORM images (Error map), equivalent FRC map (FRC map), overlay between STORM and FRC map (Overlay). Scale bar 5 μm. Figure S3 Fourier Ring Correlation (FRC) resolution mapping and NanoJ‐SQUIRREL error maps of images acquired using SB or AF647 at different illumination regimes. A, Individual A549 IFITM1‐HA expressing cells immunolabelled with anti‐HA‐streptavidin conjugated Ab labelled with B3S9L‐ATTO550‐BHQ2, WF snapshots (WF), SB super‐resolution renderings (STORM), equivalent FRC map (FRC map), overlay between STORM and FRC map (Overlay), NanoJ‐SQUIRREL error map of intensity normalised WF and STORM images. B, Same a [file TRA-21-375-s001.docx]

**Super-Beacons: open-source probes with spontaneous tuneable blinking compatible with live-cell super-resolution microscopy**

Short title: Super-Beacon probes for super-resolution microscopy

Pedro M. Pereira^1,2,4*^, Nils Gustafsson^1,5*^, Mark Marsh^1^, Musa M. Mhlanga^4^, Ricardo Henriques^1,2,#^

**Supplemental Information**

Figure S1 Super-Beacons structural, thermal and chemical tunability. a) On time ratio r = τ_ON_ ⁄ τ_OFF_, against illumination intensity, where τ_ON_ and τ_OFF_ are the weighted means of the on- and dark-state distributions respectively. (Red line) B3S9L-ATTO647N-BHQ2. (Black line) B3S9L-ATTO647N. Solid lines correspond to imaging performed in PBS; dashed lines correspond to imaging performed in switching buffer OxEA. b) On time ratio r = τ_ON_ ⁄ τ_OFF_, against illumination intensity, where τ_ON_ and τ_OFF_ are the weighted means of the on- and dark-state distributions respectively. (Red line) B3S9L-ATTO647N-BHQ2. (Black line) B3S9L-ATTO647N. (Green line) B3S9L-ATTO647N-BHQ2. Imaging performed in MiliQ water at 23°C. c) same as in b) but imaging performed in PBS at 37°C.


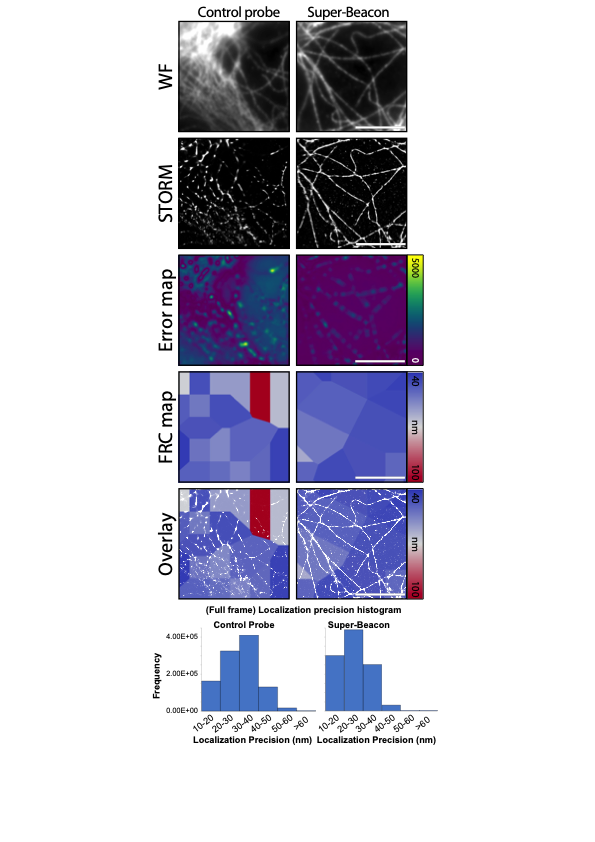


Figure S2 Fourier Ring Correlation (FRC) resolution mapping and NanoJ-SQUIRREL error maps of images acquired using SB or control probe. Left) β-tubulin immunolabelling with control probe (B3S9L-ATTO555): WF snapshots (WF), super-resolution renderings (STORM), NanoJ-SQUIRREL error map of intensity normalized WF and STORM images (Error map), equivalent FRC map (FRC map), overlay between STORM and FRC map (Overlay). Right) β-tubulin immunolabelling with B3S9L SB probe (B3S9L-ATTO555-BHQ2): WF snapshots (WF), super-resolution renderings (STORM), NanoJ-SQUIRREL error map of intensity normalized WF and STORM images (Error map), equivalent FRC map (FRC map), overlay between STORM and FRC map (Overlay). Scale bar 5 μm.


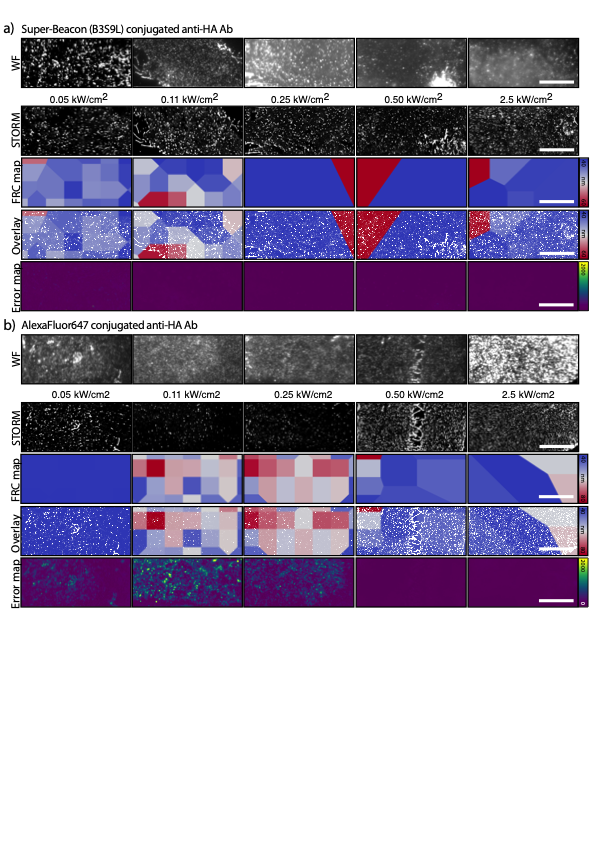


Figure S3 Fourier Ring Correlation (FRC) resolution mapping and NanoJ-SQUIRREL error maps of images acquired using SB or AF647 at different illumination regimes} a) Individual A549 IFITM1-HA expressing cells immunolabelled with anti-HA-streptavidin conjugated Ab labelled with B3S9L-ATTO550-BHQ2, WF snapshots (WF), SB super-resolution renderings (STORM), equivalent FRC map (FRC map), overlay between STORM and FRC map (Overlay), NanoJ-SQUIRREL error map of intensity normalized WF and STORM images. b) Same as in a) but for AF647 labelled Ab. Scale bars, 10 μm.
